# Complex Gastroesophageal Reflux Disease

**DOI:** 10.1016/j.gastha.2022.02.014

**Published:** 2022-04-05

**Authors:** Diana L. Snyder, David A. Katzka

**Affiliations:** 1 Gastroenterology, Mayo Clinic, Rochester, Minnesota; 2 Gastroenterology, Columbia University, New York, New York

**Keywords:** Gastroesophageal Reflux Disease, Proton Pump Inhibitor, Functional Heartburn, Achalasia, Impedance

## Abstract

Gastroesophageal reflux disease (GERD) is the most prevalent gastrointestinal disorder posing diagnostic and therapeutic challenges. Diagnosis should be objectively defined with endoscopy and pH testing, while novel metrics may augment diagnosis for inconclusive GERD cases, including the postreflux swallow-induced peristaltic wave index and esophageal mucosal impedance. Conditions that overlap with or mimic GERD should be considered such as achalasia, rumination, and eosinophilic esophagitis. Genetic testing for proton pump inhibitor metabolism is an option for precision therapy in complex persistent GERD. Proton pump inhibitor refractory GERD may require medical, surgical, or endoscopic therapies. The presence of GERD should be objectively evaluated in achalasia patients treated with peroral endoscopic myotomy, and further studies are needed to determine timing of this evaluation. Patients with scleroderma are at a high risk for GERD owing to abnormal esophageal motility and should be managed with aggressive medical therapy and lifestyle changes given the high prevalence of esophagitis and Barrett’s esophagus in this population. Further studies are needed to understand the complex mechanisms of GERD in idiopathic pulmonary fibrosis and lung transplantation.

## Introduction

Gastroesophageal reflux disease (GERD) is defined as reflux of gastric contents into the esophagus, leading to symptoms and/or pathologic complications.^1^ It is the most prevalent gastrointestinal disorder present in 8%–33% of the population worldwide and 18.1%–27.8% in North America.^2^ Symptoms alone are limited in their ability to confirm a GERD diagnosis. Patients with esophageal diseases that share common features and/or mimic GERD may present with heartburn and regurgitation. It is thus not surprising that expert clinical evaluation by a gastroenterologist is only 67% sensitive and 70% specific for GERD.^3^ Furthermore, the lack of a single diagnostic gold standard makes GERD a complex condition requiring a combination of compatible findings such clinical symptoms, endoscopic evaluation, pH monitoring, and response to therapy to confirm the diagnosis.^4^

Variability in GERD presentation and inconsistent treatment response can be due to multiple underlying mechanisms, including lower esophageal sphincter (LES) dysfunction, impaired esophageal peristalsis, impaired esophageal mucosal integrity, delayed gastric emptying, and reflux hypersensitivity.^5^ Further research is needed to elucidate the complex interaction between chemical, mechanical, and sensory mechanisms of GERD to guide future precision-directed therapy.^5^

This review will focus on complex GERD defined as GERD with unique diagnostic and management challenges. This includes defining GERD when standard diagnostics are inconclusive, a practical approach to proton pump inhibitor (PPI) refractory symptomatic and/or injurious GERD, and finally GERD as a comorbid condition or complication of treatment in complex disease states including treated achalasia, scleroderma (SSc), idiopathic pulmonary fibrosis (IPF), and lung transplantation.

## Diagnostic Dilemma of Defining the Disease

Historically, GERD has been defined by the presence of typical esophageal symptoms (heartburn and regurgitation) and an assessment for symptomatic response to empirically prescribed PPIs or other medical therapies. Owing to the heterogeneity of esophageal disorders that may present with heartburn and/or regurgitation, a more objective approach is needed to determine if symptoms are secondary to GERD or an alternate condition. According to the Lyon Consensus, objective testing is indicated for treatment failure, for an unclear diagnosis, or for treating or preventing GERD complications.^6^ The primary methods for objective testing include an upper endoscopy, wireless pH capsule, and catheter-based multichannel intraluminal pH-impedance testing.^7^, ^8^, ^9^ An important diagnostic decision is whether to perform pH and impedance monitoring on or off therapy. In general, wireless pH monitoring is recommended off therapy and catheter-based therapy, on PPIs. The latter is commonly used to demonstrate the presence of active GERD despite high-dose PPI therapy, that is, refractory GERD.^10^ Owing to the complexity of establishing a GERD diagnosis, the Lyon Consensus set out to formulate clear international recommendations for defining GERD. The consensus concluded that acid exposure time >6.0%, Los Angeles (LA) grade C or D esophagitis, long segment Barrett’s esophagus, or the presence of peptic esophageal stricture indicate clear evidence of pathologic GERD, while acid exposure time <4% with less than 40 reflux episodes provides strong evidence against GERD.^6^ The validity of the Lyon criteria has been established in a trial of nearly 500 patients (Frazzoni L., et al. 2021, Unpublished). Finally, the presence of LA grade A and B esophagitis or acid exposure time of 4%–6% is inconclusive for GERD.^6^ This has created another problematic group of patients previously diagnosed definitively with evidence of GERD.

### Novel Metrics

**Figure 1:**
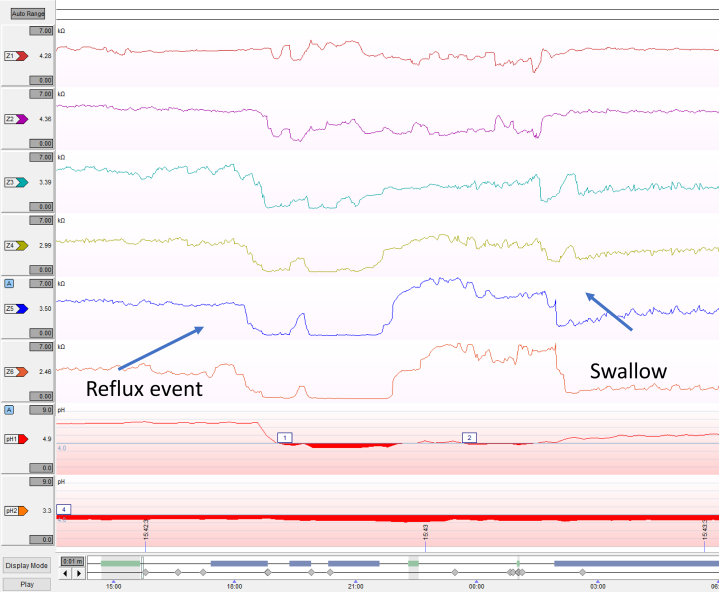
Examples of reflux events with the presence or absence of postreflux swallow-induced peristaltic waves.

Owing to the larger population of patients that fall into the inconclusive for GERD category, additional objective evidence is needed to establish a clear diagnosis. Novel metrics that may enhance a GERD diagnosis in the setting of equivocal findings on pH testing and endoscopy are the postreflux swallow-induced peristaltic wave (PSPW) index, baseline impedance, and mucosal impedance. The PSPW index is defined as the proportion of reflux episodes on pH-impedance testing that are followed by a PSPW (Figure 1). A value of 61% of intact PSPWs is considered the normal cutoff.^11^ A low index has a 99%–100% sensitivity and 92% specificity for differentiating pathologic acid exposure from functional heartburn or healthy controls. This is currently being used for research purposes and needs to be manually calculated on pH-impedance software.^12^^,^^13^

**Figure 2:**
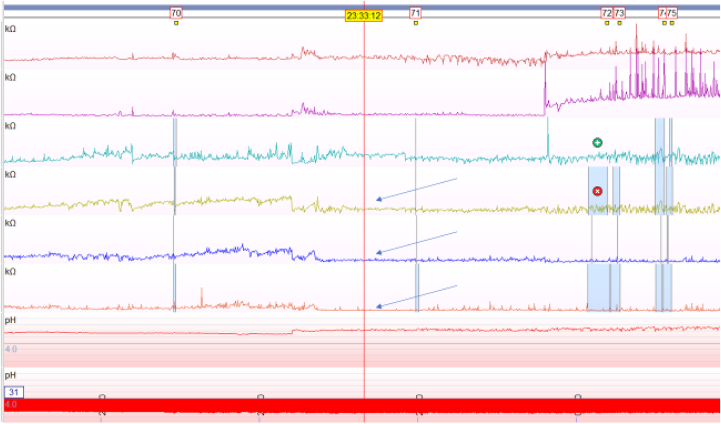
Low baseline nocturnal impedance in the distal 3 esophageal impedance leads.

A second novel diagnostic metric is baseline impedance. Impedance is defined as resistance to electrical flow, and low baseline impedance is present in patients with erosive or nonerosive GERD owing to changes in mucosal permeability.^14^^,^^15^ Baseline impedance may be measured during sleep through mean nocturnal baseline impedance (MNBI) or during high-resolution esophageal impedance manometry (HRIM) (Figure 2). MNBI is lower in erosive and nonerosive reflux disease than that in functional heartburn and healthy controls.^12^^,^^16^^,^^17^ The advantage of nocturnal MNBI measurement is reduced interference of frequent swallowing and reflux events during sleep; however, this is a cumbersome process requiring an average of 3 nocturnal 10-minute baseline periods.^12^^,^^18^ More recently, a simplified MNBI analysis method using automatic software calculations over the entire supine period provided similar results to the conventional analysis.^19^ Another more streamlined approach is to utilize baseline impedance measurements over 15 seconds during the landmark period of HRIM. In a recent study, HRIM baseline impedance below 1582 Ω had a sensitivity of 86.2% and specificity of 88.5% for GERD.^20^ Notably, the precise normal baseline value for mucosal impedance is variable among studies with a more recent study using >2292 ohms (Ω) as normal.^11^ A 2020 international consensus noted the MNBI below 1500 Ω suggests impaired esophageal mucosal integrity.^21^

Finally, a third diagnostic tool that may aid in the complex diagnosis of GERD is pan-esophageal mucosal impedance. This method of mucosal impedance technology uses a balloon catheter with 36 channels to measure impedance over 10 cm of the esophagus during endoscopy. A multicenter prospective study found a significant difference in mucosal impedance between patients with GERD, with eosinophilic esophagitis (EoE), and without GERD. Patients with GERD have low impedance in the distal esophagus that transitions to normal proximally, while EoE patients exhibited low impedance throughout the esophagus. Non-GERD patients had higher mucosal impedance values along the entire measured esophageal segment.^22^ This new device may be particularly useful in distinguishing GERD from EoE given the overlap in clinical presentation and patchy distribution of eosinophils that may be missed on endoscopic biopsies. Further study is needed to determine reproducibility and generalizability of these results.

## PPI Refractory GERD

**Figure 3:**
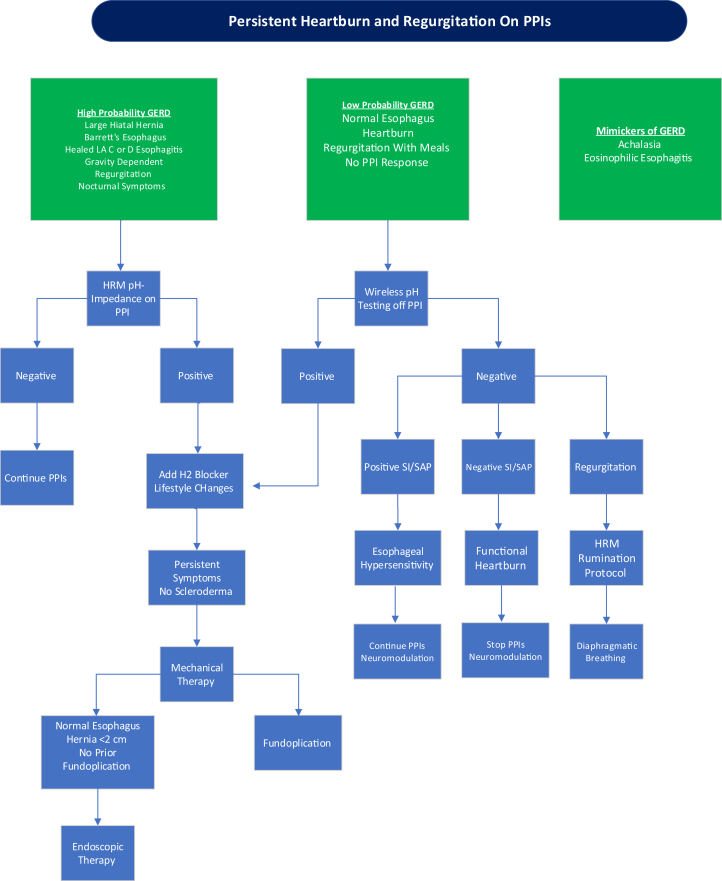
Approach to heartburn and regurgitation that do not respond to proton pump inhibitors.

Up to 40% of patients with suspected GERD do not have adequate response to PPI therapy.^23^ This may be due to inadequate acid suppression, nonacid reflux, or alternative diagnoses (Figure 3). PPI refractory GERD is defined by objective evidence of GERD with a lack of adequate symptomatic response, persistence of abnormal acid exposure, and/or GERD complications in the presence of PPI therapy. Although heartburn and regurgitation are common symptoms of GERD, multiple esophageal disorders have similar symptoms which can be challenging to distinguish from GERD with careful history taking alone. Furthermore, some esophageal disorders may share overlapping physiology with GERD. Thus, it is important to investigate other etiologies when patients are referred for inadequate response to PPIs before considering PPI refractory GERD. These disorders include achalasia, rumination syndrome, supragastric belching, EoE, and functional heartburn. It is also important to exclude nonesophageal conditions such as cardiac disease.

### Evaluation for Non-GERD Causes of Refractory Symptoms

Achalasia is a primary esophageal motility disorder due to esophageal myenteric plexus damage, leading to loss of peristalsis and impaired esophagogastric junction relaxation.^24^ A careful history assessment is needed to increase clinical suspicion that chest pain and regurgitation are secondary to achalasia though heartburn without dysphagia may be an early presenting symptom.^25^ Whether this is an early symptom of esophageal distention and stasis is unclear. Appropriate testing should be initiated including timed barium esophagram and HRIM and before proceeding with management for refractory GERD.^26^ Moreover, it is paramount to exclude this diagnosis before considering antireflux surgery since this could result in worsening dysphagia.^4^

Rumination syndrome is a disorder of unclear etiology which manifests as effortless regurgitation of recently swallowed food from the stomach into the mouth followed by reswallowing or spitting. This condition is important to recognize since the effortless regurgitation of food may be mistaken for regurgitation in GERD. Similarly, rumination may coexist in patients with objective GERD. A study of patients with PPI nonresponsive esophageal symptoms found 20% had evidence of rumination on HRIM.^27^ Another study evaluating ambulatory multichannel intraluminal impedance-pH monitoring found postprandial rumination is found in 46% of patients with refractory GERD as suggested by a marked increase in immediate postprandial nonacid reflux events.^28^ Rumination is diagnosed based on Rome IV criteria in the setting of a postprandial HRIM study, showing increased gastric pressure events (an R wave) with movement of impedance to the proximal esophagus.^29^^,^^30^ Adding to the complexity of defining these diagnoses, recent evidence found postprandial gastric pressure is significantly higher in patients with pH-proven GERD than that in controls, suggesting overlap in pathophysiologic mechanisms of GERD and rumination and lowered with diaphragmatic breathing. A randomized controlled trial found that postprandial diaphragmatic breathing significantly reduces the number of reflux events in patients with upright GERD; therefore, diaphragmatic breathing may also serve as an adjunct therapy for GERD.^31^

**Figure 4:**
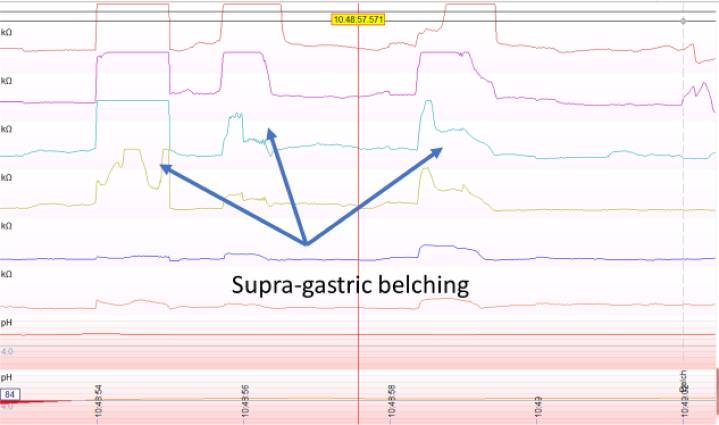
Supragastric belching identified on impedance testing.

Supragastric belching is another condition that should be identified in patients initially suspected to have GERD, but who have poor response to PPIs. It is a pathologic condition in which air enters and leaves the esophagus quickly without reaching the stomach.^32^ Supragastric belching is distinguished from physiologic gastric belching using impedance-pH monitoring (Figure 4). A study of patients presumed to have GERD with PPI nonresponsiveness found that 42% had supragastric belching on esophageal impedance testing.^27^ Supragastric belching is important to define since it responds to behavioral therapies rather than acid suppression.^32^

EoE is another esophageal disorder that may present with similar symptoms to GERD. About 30%–60% of patients with EoE have heartburn which could be secondary to the inflammatory mucosal response.^33^^,^^34^ This appears more likely in older children and teenagers. Furthermore, GERD and EoE may coexist in patients, requiring esophageal biopsies and pH testing for evaluation.

Reflux hypersensitivity and functional heartburn should also be distinguished from GERD through objective testing. Patients with reflux hypersensitivity have heartburn that correlates with physiologic reflux events, which can be considered a unique phenotype on the GERD spectrum. It is defined as normal acid exposure time in the setting of a positive symptom index and/or symptom association probability on pH testing with exclusions of esophagitis, EoE, or a major motility disorder on HRIM.^35^ Functional heartburn or chest pain is a diagnosis of exclusion defined as heartburn in the setting of normal acid exposure on pH testing without symptom correlation, normal esophagogastricduodenoscopy with no histologic abnormalities, and no evidence of a major motility disorder on HRIM.^36^ It is common in patients presenting with refractory symptoms. A recent randomized trial evaluating medical vs surgical management for refractory heartburn found that 27% of patients initially enrolled were ultimately excluded since they were diagnosed with functional heartburn after objective testing.^37^ Neuromodulators are considered important treatment for reflux hypersensitivity, but data are needed to support this. There are randomized placebo-controlled trials supporting use of neuromodulators in reducing functional heartburn or chest pain.^38^

### Management Strategies for PPI Refractory GERD

Once non-GERD etiologies are excluded, the initial management approach is to educate patients on lifestyle measures including avoiding meals 3 hours before the supine position, elevation of the torso in bed, and achieving and maintaining a healthy body mass index (BMI). In addition, it is important to optimize PPI dosing and confirm appropriate administration 30–60 minutes before meals. The greatest therapeutic response of PPIs is healing of erosive esophagitis followed by control of heartburn.^39^ The American College of Gastroenterology guidelines support only one empiric change in the PPI if there is lack of response to an initial PPI.^4^ Precision medicine may be used to optimize PPI therapy. Genetic variants in the gene that encodes the CYP450 2C19 isoenzyme can alter PPI metabolism. Patients with rapid or ultra-rapid metabolism through this pathway may benefit from treatment with rabeprazole which is primarily metabolized through a nonenzymatic pathway.^40^

For GERD symptoms due to other mechanisms, adjunctive medical therapies should be considered. Histamine-2 receptor antagonists may be used for control of nighttime symptoms although sustained efficacy is limited by tachyphylaxis.^41^ Gamma amino butyric acid agonists such as baclofen can reduce the frequency transient lower esophageal relaxations but may be limited by neurologic side effects and/or fatigue.^42^ Sodium alginate preparations are useful for postprandial symptoms by neutralizing the gastric “acid pocket and providing esophageal mucosal protection.”^43^ Potassium-competitive acid blockers such as vonoprazan which work with less inhibition through CYP2C19 may be future therapies, but are not currently approved in the United States.^44^

Surgical or endoscopic approaches may be used in patients with objectively confirmed GERD who do not respond to lifestyle measures and medical therapy. The nature of refractory disease can range from persistent mucosal injury such as erosive esophagitis or refractory GERD strictures to uncontrolled symptoms. One problematic symptom of GERD is regurgitation. Studies using sphincter-directed mechanical therapy such as with a laparoscopic magnetic sphincter augmentation device or transoral incisionless fundoplication (TIF) have demonstrated a significantly greater response of regurgitation than those using PPIs.^45^, ^46^, ^47^ Furthermore, a meta-analysis has demonstrated only a 50% overall response to PPIs.^48^ This is not surprising as regurgitation likely represents complete LES failure that persists in the presence of complete acid suppression. The performance of sphincter repair or augmentation therapy for control of Barrett’s metaplasia is more controversial. Although there is good logic and some clinical trials demonstrating that optimal control of reflux may attenuate the risk of developing adenocarcinoma, no clear superiority has been demonstrated of fundoplication over PPI therapy.

Surgical approaches to refractory complex GERD include laparoscopic fundoplication and Roux-en-Y gastric bypass surgery. A 2011 5-year randomized multicenter European trial found that standardized laparoscopic antireflux surgery maintained GERD remission in 85% of patients at 5 years.^49^ In 2019, a randomized trial evaluating management for refractory heartburn reported that in the select group with confirmed refractory heartburn, surgical therapy was highly successful in 67% of patients.^37^ The recent American College of Gastroenterology guidelines recommend antireflux surgery when performed by an experienced surgeon in patients with objective evidence of GERD.^4^ Patients with LA grade C or D esophagitis, large hiatal hernias, and/or persistent GERD symptoms are most likely to benefit.^4^ It is optimal to heal the esophagitis if possible and avoid the periesophageal inflammation during surgery associated with persistent erosive disease. Preoperative HRIM is required before antireflux surgery, and evaluation of esophageal reserve using the multiple rapid swallow sequence may help predict which patients are likely to have late postoperative dysphagia.^50^ More specifically, patients with ineffective esophageal motility may benefit from a less than 360° wrap though this is controversial and ineffective esophageal motility is variably defined based on the time of publication. In patients with obesity, Roux-en-Y gastric bypass is preferred as an antireflux therapy that will also treat other comorbidities.^51^ This approach is taken to avoid a more rapid and higher incidence of fundoplication failure in patients with obesity. The cutoff BMI with which to stratify patients for bypass or fundoplication is unclear but is generally 30–35. Perhaps, a more accurate determinant would be a measure of central obesity.

Owing to adverse symptoms such as dysphagia and bloating and the abdominal approach associated with laparoscopic antireflux surgery, endoscopic antireflux techniques have been further evaluated.^49^ TIF is a recent endoscopic procedure that may be considered as an alternative to surgical fundoplication. It can be performed in select patients without a significant hiatal hernia and diaphragmatic crural defect, erosive esophagitis, or Barrett’s esophagus. During this procedure, a retractor is used to position the distal esophagus below the diaphragm, while the gastric fundus is folded around the distal esophagus and plicated with fasteners to form a 270° partial fundoplication.^52^ A few randomized controlled trials have evaluated the most recent TIF 2.0 technique. One noted troublesome regurgitation was resolved in 97% of patients who completed TIF compared to 50% of patients on the PPI at 6-month follow-up. Of the 63 patients in the trial who received TIF, 44 were evaluated up to 5 years with 86% having elimination of troublesome regurgitation.^46^ Another sham-controlled trial found TIF eliminated regurgitation in 67% of patients compared to 45% on once or twice daily omeprazole at 6 months. Secondary analysis noted improvement in heartburn.^47^ Further study is needed to determine longer-term outcomes including rates of patients who remain PPI free after TIF. TIF combined with laparoscopic hernia repair is currently under investigation.^52^

## GERD Evaluation and Management in the Setting of Complex Disease States

Management of GERD in the setting of other complex disorders proposes further challenges including achalasia after peroral endoscopic myotomy (POEM), SSc, IPF, and lung transplantation.

### Achalasia After Peroral Endoscopic Myotomy

Durable therapies for achalasia disrupt the LES in order to improve esophageal emptying.^24^ Laparoscopic Heller myotomy (LHM), POEM, and pneumatic dilation are effective treatments for achalasia.^24^^,^^53^ While opening the esophagogastric junction facilitates food and liquid transit into the stomach, it can also enable retrograde flow and, hence, GERD. For LHM, a modified fundoplication provides some protection against reflux with GERD rates in LHM ranging from 11% to 32%.^54^, ^55^, ^56^, ^57^ Thus far, an antireflux procedure has not been performed with POEM with the assumption that the technique minimizes disruption of the reflux barrier including the hiatus and angle of His.^58^ Nevertheless, reflux is a prevalent problem after POEM. This is not surprising as LES disruption in the presence of absent esophageal clearance is a model for GERD. On pH testing after POEM, acid exposure time is abnormal in about 20%–53% of patients.^55^^,^^56^^,^^58^ Erosive esophagitis is found in 16%–57%, with most patients having LA grade A or B and responsive to medical therapy.^58^, ^59^, ^60^ There is no difference in GERD rates based on an anterior vs posterior myotomy in POEM.^59^^,^^60^ Furthermore, achalasia patients with post-treatment GERD can be asymptomatic; therefore, objective measurement with endoscopy and pH testing is important to identify candidates for GERD management and avoid complications.^61^, ^62^, ^63^ Further study is needed to determine the ideal timing of pH testing after POEM, and it is imperative to counsel patients on the comparative risk of GERD before proceeding with therapy.

### Scleroderma

Patients with systemic sclerosis (SSc) have frequent heartburn and regurgitation.^64^, ^65^, ^66^ SSc affects the esophagus through injury to the myenteric plexus through antimyenteric neuronal antibodies and abnormal collagen deposition secondary to diffuse damage of small vessels. This results in fibrosis and ultimately impaired smooth muscle contractility.^67^, ^68^, ^69^ Diminished function of the LES and esophageal body predisposes SSc patients to significant GERD. Dysfunction of the LES occurs in 50% of SSc patients, leading to a hypotensive esophagogastric junction pressure with hypofunction of the reflux barrier. In addition, aperistalsis in the lower smooth muscle segment of the esophagus impairs esophageal clearance of injurious gastric refluxate.^70^ SSc patients are at a high risk for GERD complications. Studies report that up to 60% of SSc patients have erosive esophagitis.^71^, ^72^, ^73^, ^74^ The prevalence of Barrett’s esophagus in the SSc population is higher than that in the general population estimated at 12.8%. It is important that SSc patients receive adequate PPI therapy to reduce acid exposure and progression of dysplasia.^75^ This often requires multiple daily doses of medication and lifestyle precautions. Fundoplication is generally contraindicated with scleroderma esophagus because of the high incidence of severe dysphagia following sphincter augmentation in the presence of esophageal aperistalsis.

### Idiopathic Pulmonary Fibrosis

IPF is a chronic progressive lung disease of unclear etiology. The morbidity and mortality of IPF are high; therefore, identifying appropriate causes may guide management. The association between GERD and pulmonary fibrosis is not fully defined. It is hypothesized that GERD may lead to chronic microaspiration with recurrent lung injury eventually causing pulmonary fibrosis. Alternatively, patients with pulmonary fibrosis have increased negative thoracic pressure that can alter function of the lower and upper esophageal sphincters, leading to microaspiration through a vacuum effect. Further evaluation is needed to define these mechanisms.^76^ Studies have shown a higher prevalence of GERD in the IPF population than in the general population with up to 94% of IPF patients having abnormal acid exposure on 24-hour pH testing but only about 47% exhibiting classic GERD symptoms.^77^^,^^78^ In addition, an increasing incidence of GERD in IPF patients is reported.^79^ In contrast, a recent meta-regression analysis of 18 case-control studies did not find a correlation between IPF and GERD once the data were controlled for smoking.^80^ Studies suggest that PPIs stabilize lung function and increase survival in IPF; thus, current IPF guidelines provide a conditional recommendation to use antireflux therapies for patients with IPF.^77^^,^^79^^,^^81^^,^^82^ A difficult question arises when there is progressive IPF in the presence of proven uncontrolled GERD, and fundoplication is recommended. Further studies are needed to investigate the association between GERD and IPF in addition to better defined use of medical and surgical management strategies.

### Lung Transplantation

In addition to its role in lung function decline for patients with chronic lung disease such as IPF, GERD also impacts lung transplant outcomes. The prevalence of GERD is very high in patients with end-stage pulmonary disease being evaluated for lung transplant with abnormal 24-hour pH testing in up to 68% of patients.^83^ GERD management has important implications for lung transplant outcomes. Chronic allograft rejection or bronchiolitis obliterans syndrome after lung transplant requires a complex interaction of immunologic and nonimmunologic processes. It is hypothesized that GERD may contribute to the nonimmunologic pathway through aspiration of gastroesophageal contents, leading to transplant graft injury.^84^ A retrospective study determined equivalent safety outcomes for laparoscopic antireflux surgery in lung transplant recipients compared to patients without lung transplant.^85^ Multiple studies have evaluated antireflux surgery and allograft function. A recent meta-analysis of 6 studies found that patients with declining forced expiratory volume in 1 second may benefit from antireflux surgery.^86^ The role of PPIs is less clearly defined. A retrospective study of 188 lung transplant recipients noted reduced rates of allograft rejection for patients with PPI acid suppression independent of the BMI.^87^ Further study is needed on medical acid suppression in lung transplant patients.

## Conclusions

GERD is a prevalent disorder that poses complex diagnostic and therapeutic challenges. Objectively defining the disease is imperative since other esophageal disorders may overlap in clinical presentation. Novel metrics including the PSPW index, baseline impedance, and mucosal impedance may guide diagnosis. PPI refractory GERD should be defined with objective testing, and management strategies include genetic testing for ultra-rapid metabolism, medical therapies, and surgical or endoscopic management. Further studies are needed to guide management of GERD in complex disease states such as achalasia after POEM, SSc, IPF, and lung transplantation.
